# Automated Daily Phone Surveys in Older Adults: Feasibility Study in the Multi-Ethnic Study of Atherosclerosis

**DOI:** 10.2196/86333

**Published:** 2026-07-16

**Authors:** Sally J Kim, Cyanna McGowan, Shaina J Alexandria, Grace D Achepohl, Lauren Hoffer, Norrina B Allen, Mandy Wong, Kiarri N Kershaw

**Affiliations:** 1 Department of Preventive Medicine, Northwestern University Feinberg School of Medicine, 680 North Lake Shore Drive, Suite 1400, Chicago, IL, 60611, United States, 1 312-908-7914

**Keywords:** ecological momentary assessment, older adults, daily diary, feasibility, phone-based survey, aging research, Multi-Ethnic Study of Atherosclerosis

## Abstract

**Background:**

An ecological momentary assessment (EMA) is a methodological framework designed to capture real-time data in a participant’s natural environment, yet its feasibility in older adults, particularly in a large, multisite study, remains underexplored.

**Objective:**

This study aims to assess the feasibility of implementing automated daily phone surveys in a large, geographically diverse cohort of older adults.

**Methods:**

A total of 1283 participants from the Multi-Ethnic Study of Atherosclerosis (MESA) were enrolled across 6 US field sites. Participants were scheduled to receive 1 automated phone survey per day for 7 consecutive days following a clinic visit. Feasibility was assessed through a dual lens of participant engagement (survey adherence, phone survey duration, and cancellations) and administrative implementation (scheduling errors).

**Results:**

Of the 1272 participants who received at least 1 of the 7 phone surveys, high adherence was observed, with 81.84% (1041/1272) completing 5 to 7 surveys. Age was the only demographic characteristic significantly associated with completion (*P*=.002), as younger participants demonstrated higher adherence. While the median survey duration was 3 (IQR 2‐4) minutes, 26.84% (2371/8835) of surveys lasted 5 minutes or longer. However, adherence remained remarkably robust even among participants experiencing these extended durations. Participant-initiated cancellations during the 7-day monitoring period were rare, occurring among 21 of 1272 (1.65%) participants who began monitoring. Administrative scheduling errors were also uncommon, accounting for 37 of the 8835 (0.42%) total scheduled surveys not being delivered.

**Conclusions:**

Findings support the feasibility of automated daily phone surveys for collecting repeated real-world data in large, multisite studies of older adults. Future studies should incorporate standardized participant feedback to identify barriers to engagement and support more inclusive, participant-centered adaptations of automated phone-based EMA protocols.

## Introduction

Ecological momentary assessment (EMA) encompasses a diverse methodological framework designed to capture real-time data on thoughts, feelings, and behaviors within the context of a participant’s natural environment. By using repeated sampling, EMA offers a more dynamic picture of daily life that effectively minimizes the recall biases often associated with traditional laboratory-based or retrospective methods [1]. EMA exists on a spectrum regarding its procedural flexibility: sampling protocols can be structured as signal-contingent (random prompts), event-contingent (participant-initiated), or interval-contingent (fixed schedules). The frequency and duration of EMA vary significantly across research designs; protocols may range from multiple assessments within a single day to a single assessment over many days, and monitoring periods are similarly variable in length [2-5]. These assessments can also be deployed through a wide variety of interfaces, ranging from mobile apps and wearable sensors to paper diaries and telephone-based systems [1,6-8].

While older adults have been increasingly included in EMA research and recent research suggests that EMA compliance in older adults is generally promising, the broader body of evidence remains mixed and limited [9-18]. A 2023 systematic review and meta-analysis by Yao et al [17] indicates a combined compliance rate of 86.41%, yet completion rates are significantly influenced by participant-level factors and much of the existing evidence is limited by methodological constraints. Studies are predominantly characterized by small sample sizes, often less than a sample size of 100, and limited multisite recruitment [9-18]. Consequently, while existing research provides a foundational understanding of EMA feasibility, these findings may not fully generalize to the unique complexities of studies involving a larger, geographically diverse sample of older adults.

To address these limitations and accommodate the significant scale and geographic distribution of the Multi-Ethnic Study of Atherosclerosis (MESA), we implemented an EMA framework designed for both participant acceptability and operational sustainability. Specifically, among the diverse sampling strategies categorized under EMA, we used the daily diary approach, which is a fixed-interval method involving a single assessment at a consistent time each day [1]. This method was selected to support large-scale implementation because its fully automated, phone-based interface enabled data collection across a multisite cohort of more than 1000 older adults while minimizing burden on participants and research staff.

To evaluate the feasibility of this approach, we used a dual lens that considered both participant engagement and the administrative requirements of the study. Participant engagement feasibility reflected whether participants completed and continued with the daily phone surveys. Participant engagement was evaluated through descriptive participant characteristics, survey adherence metrics, and participant-initiated cancellation of the phone surveys. These measures allowed us to characterize the stress reactivity ancillary study sample relative to the broader MESA Exam 7 cohort, identify patterns across survey adherence groups, describe the extent to which participants completed the phone surveys and their experience, and assess active requests to stop future phone surveys. Administrative implementation feasibility was assessed by quantifying scheduling errors that disrupted the full delivery of phone surveys during the 7-day monitoring period. Specifically, we tracked the number of participants impacted by at least one scheduling error and the total volume of missed surveys relative to the overall study target. This study aims to assess the feasibility of automated daily phone surveys in a large, multisite study of older adults. Our findings contribute to the growing body of work on EMA use in older adults and offer practical guidance for implementing automated phone-based protocols in large-scale, multisite studies.

## Methods

### Study Population

The MESA is an ongoing, community-based cohort study designed to investigate the prevalence and progression of subclinical cardiovascular disease among adults. The study enrolled 6814 racially and ethnically diverse men and women aged 45 to 84 years who were free of known cardiovascular disease at enrollment. Participants were recruited from 6 field centers across the United States: New York, NY; Baltimore, MD; Forsyth County, NC; St. Paul, MN; Chicago, IL; and Los Angeles County, CA. Baseline (Exam 1) data collection took place from 2000 to 2002, with multiple examinations conducted thereafter among participants from Exam 1: Exam 2 (2002‐2004), Exam 3 (2004‐2005), Exam 4 (2005‐2007), Exam 5 (2010‐2011), Exam 6 (2016‐2018), and Exam 7 (2022‐2024). These participants were followed longitudinally to identify updates on health, interim hospitalizations, cardiovascular events, and mortality. Detailed information on the MESA study and recruitment procedures has been published previously [19] and is available on the study website [20].

Exam 7 consisted of core examination components and 7 ancillary studies. Participants from the original MESA cohort who remained active at follow-up were invited to complete the core Exam 7 visit. All participants completing the core exam were considered for ancillary studies unless they met ancillary-specific exclusion criteria. A total of 2276 MESA participants successfully completed Exam 7 [20]. Of this initial cohort, 1834 individuals were identified as eligible for recruitment into ancillary studies. Among those eligible, 1524 participants provided informed consent to participate in the ancillary studies. Within this consenting group, 1283 individuals agreed to participate and were enrolled specifically in the MESA Stress Reactivity Ancillary Study (ancillary study). Reasons for attrition included ineligibility at the screening phase, declining to participate in ancillary research generally, or declining the specific protocols required for the stress reactivity ancillary study.

This paper primarily focuses on participants and data from the MESA Stress Reactivity Ancillary Study. Participants were eligible if they agreed to wear a continuous ambulatory heart monitor (Cardiac Insight, Inc) and complete daily phone-based surveys over a 7-day monitoring period. Exclusion criteria included allergy to skin adhesives, refusal to shave the upper left chest, and the presence of implanted electronic devices such as pacemakers. The stress reactivity ancillary study component of the Exam 7 visit was estimated to take 30 minutes and included consent, heart monitor application, and participant instructions. Although enrollment in the stress reactivity ancillary study included both heart monitor wear and daily phone surveys, the present analysis focuses specifically on the feasibility of implementing the automated daily phone survey protocol.

### Automated Daily Phone Surveys Protocol

Given the large, multisite structure of MESA and the average age of its participant population (mean 77.3, SD 7.50 years), selecting an EMA method that balanced acceptability, scalability, and staff sustainability was a priority. To gauge possible data collection options, an off-cycle survey covering a variety of topics was administered to MESA participants. Among the 1381 respondents, 39.03% (539/1381) indicated willingness to use a mobile device for data collection. Given the limited interest in using mobile devices for data collection in the larger MESA cohort, an alternative EMA delivery method was needed as opposed to smartphone app-based EMA.

Our study drew on Almeida’s daily telephone interview approach [21], in which participants completed brief telephone interviews that took approximately 10 to 15 minutes each evening for 8 consecutive days. However, for the stress reactivity ancillary study, live interviewer-administered calls were not considered practical considering the number of participants in MESA. Additionally, since the willingness to use mobile devices for data collection was low, an alternative approach that did not depend on mobile device use was needed. This led to the selection of a call-based method that could also accommodate participants who relied on landlines. Collectively, these considerations led to a modified version of the Almeida approach that used a centralized, automated system to deliver the daily phone surveys.

The automated phone-based system was implemented using REDCap, a data collection and management tool developed by Vanderbilt University, together with the third-party voice delivery service Twilio Voice (Twilio Inc). REDCap was used to store participants’ phone survey scheduling preferences, daily stress survey questions, and responses, while Twilio served as the platform for generating automated outbound calls. At the clinic visit (day 0), field center staff entered the information needed to schedule the automated daily phone surveys in REDCap, including the date of the first call, the participant’s preferred call time, preferred language (English, Spanish, Mandarin, or Cantonese), time zone, and phone number. This 1-time entry triggered the automated survey call sequence for the full 7-day monitoring period, so that the field center staff did not need to manually reschedule calls for each day of the monitoring period. This initial entry was critical because it governed both the first scheduled call and the subsequent daily calls over the 7-day monitoring period. Incorrect entry could therefore disrupt the delivery of the full survey sequence and affect participants’ opportunity to receive or complete surveys.

The first call was intended to be scheduled for the day after the clinic visit. Participants selected a preferred call time after 4:00 PM based on their availability, as this time window was intended to increase the likelihood that participants would be at home and available to answer the phone surveys if they indicated to receive the daily phone surveys at their landline. The preferred call time had to be entered into REDCap in military format for the automated calls to go out after 4:00 PM. For instance, if a clinic visit occurred on January 1, 2023, and the participant selected 4:30 PM as their preferred call time, the first call should be entered into REDCap as January 2, 2023, 16:30.

The daily stress survey was programmed in REDCap as a survey instrument, and participants heard the survey questions from this instrument during the automated phone calls delivered through the Twilio Voice integration. Therefore, once the participants’ scheduling information was entered, REDCap transmitted both the survey content and call settings to Twilio, which placed the automated call. REDCap rendered each survey question, Twilio delivered the questions by phone, participants entered responses using their phone keypad, and the responses were returned to and stored in REDCap. This process was repeated until all survey questions were completed, after which the call ended [22]. For participants who selected a language other than English, the daily stress survey questions and call script were translated from the English version and delivered in the participant’s preferred language: Spanish, Mandarin, or Cantonese. Non-English calls used text-to-speech audio through the same automated workflow using REDCap and Twilio. No additional study-specific modifications were made to the automated system for tonal languages. This approach accommodated participant language and scheduling preferences while reducing staff burden and supporting standardized data collection across multiple sites.

From the participant’s perspective, once this 1-time setup was initiated at the clinic visit by the field center staff, participants received 1 phone survey per day for 7 consecutive days, beginning the day after their clinic visit. The daily stress surveys were delivered each day at the participants’ preferred time. Participants responded to these phone-based surveys using their phone keypad. Most questions had “Yes” or “No” response options, with responses recorded as “1” for Yes and “0” for No. Some questions included branching logic, where a Yes response triggered follow-up questions answered on a numeric scale, typically ranging from 1 to 4 but extending up to 7. Each survey was expected to be completed within 5 minutes. If a participant missed the initial call, the system automatically initiated a follow-up call 15 minutes later. Up to 2 additional follow-up calls were sent at 15-minute intervals for a maximum of 3 phone calls per day across the 7-day monitoring period. If all 3 attempts were missed, the phone survey was considered incomplete for that day. Surveys were also classified as incomplete if a participant terminated the call early before responding to all survey questions. A survey was considered complete if the participant answered the call and progressed through all the programmed questions. The surveys were not designed to allow partial completion.

### Procedures to Support Survey Delivery and Protocol Adherence

Several procedures were put in place to support accurate protocol implementation and maximize the likelihood that participants received all 7 scheduled phone surveys. Participants using cell phones were encouraged to program the Twilio number into their contacts under “MESA Study” to minimize the risk of calls being missed or marked as spam. For those using landlines, the caller ID displayed “MESA Study.” During the initial clinic visit, participants also completed a demo phone survey to familiarize themselves with the automated call system. They were asked to dial a practice number and complete a brief test phone survey using the same response format as the scheduled calls, with field center staff available to answer any questions and provide assistance during this practice session.

To support accurate protocol implementation and ensure quality control, the centralized coordinating team (hereinafter “reading center”) for the stress reactivity ancillary study received real-time notifications after the REDCap scheduling forms were completed by the field center staff. Upon receiving the notification, the reading center staff reviewed the entered information for accuracy and completeness. If any discrepancies or missing data were identified, the reading center staff contacted the appropriate field center to request corrections. Once the information was corrected, the call schedule was reprogrammed accordingly via the Twilio platform. Because the first phone survey was scheduled for any time after 4:00 PM the day after the initial clinic visit, field center staff were expected to complete the scheduling form as close to the clinic visit as possible. This allowed the reading center staff enough time to review the information and request or make corrections before the first scheduled phone survey. All communication between the reading center and field centers was documented in Smartsheet (Smartsheet Inc), a centralized work management platform used to track and coordinate study operations.

Field center staff were also instructed to conduct a check-in call with participants 2 days after the clinic visit and following the first scheduled phone survey to ensure the participant had not encountered any issues with receiving or completing the survey. If the first survey was missed, field center staff were expected to work with the participants to identify the reason and provide support as needed. In parallel, the reading center staff monitored survey completion status in REDCap for each participant daily. If a participant missed 2 consecutive surveys, the reading center staff contacted the corresponding field center staff to coordinate outreach and troubleshoot ongoing issues. Participant feedback, when provided, was documented in Smartsheet. At the conclusion of the 7-day monitoring period, the total number of completed surveys per participant was tracked in REDCap.

### Feasibility

To assess the feasibility of our EMA approach, we considered both the participant experience and the administrative requirements of the study. Participant engagement feasibility was evaluated using three components: (1) descriptive participant characteristics, (2) survey adherence, and (3) participant-initiated cancellation of the phone surveys. Descriptive participant characteristics were examined for 2 purposes. First, we compared participants enrolled in the MESA Stress Reactivity Ancillary Study with MESA Exam 7 participants who were not enrolled in the ancillary study. Second, among ancillary study participants included in the adherence analysis, we examined demographic and socioeconomic characteristics across survey completion groups to explore patterns in participant engagement, with particular attention to participants with high adherence and zero adherence.

Survey adherence was defined as the number of daily phone surveys completed during the 7-day monitoring period. Participants were categorized into 4 completion groups: 0 surveys, 1‐2 surveys, 3‐4 surveys, and 5‐7 surveys. Completion of 5‐7 surveys was considered high adherence because it represented completion of most of the daily phone surveys. This categorization was informed by EMA studies that commonly define high adherence at the participant level as completion of approximately 80% of prompts, although this threshold is not formally established [13]. At the opposite end, participants with zero adherence were defined as participants completing 0 surveys. Means and SDs were reported for continuous variables, and frequencies were reported for categorical variables across the 4 completion groups. These analyses were descriptive in nature, and no formal hypothesis testing was performed. We also characterized participant engagement by analyzing survey duration, defined as the time elapsed from phone survey initiation to completion, to assess the burden and tolerability of the daily phone surveys.

In addition to survey adherence, we also assessed participant-initiated cancellation of the daily phone surveys as another indicator of participant engagement feasibility. Participant-initiated cancellations were defined as participant requests to stop the daily phone survey and were categorized as either premonitoring or during monitoring cancellations. Premonitoring cancellations were defined as requests to opt out of the phone surveys before the start of the 7-day monitoring period. During monitoring, cancellations referred to requests made after the first phone survey went out and at any point during the 7-day monitoring window. Participants could request cancellation by notifying field center staff, including at the clinic visit or after the monitoring period had begun.

Participants who canceled before monitoring began did so before the first scheduled phone survey was delivered and therefore did not receive any daily phone surveys. These premonitoring cancellations were retained as a separate subgroup because they represented participant-initiated requests to stop the phone survey component before survey delivery began, which differs from missed or incomplete surveys due to nonresponse or administrative scheduling errors. Therefore, premonitoring cancellations were not included in the survey adherence analysis. Participants who canceled after the monitoring period began were included in adherence analyses based on the number of surveys completed before cancellation. There was no standardized protocol requiring staff to systematically ask for or record reasons for cancellations. As a result, cancellation reasons were available only in some cases and were not analyzed formally. When available, these reasons were used descriptively to provide context for possible participant burden, lack of interest, or challenges with the automated phone survey system.

Administrative implementation feasibility was defined as the extent to which study staff successfully followed the automated daily phone survey protocol according to study procedures. A scheduling error was defined as any staff entry error that resulted in one or more expected daily phone surveys not being sent as intended. Therefore, to evaluate administrative implementation feasibility, we calculated (1) the number and proportion of participants affected by at least one scheduling error, and (2) the number and proportion of expected daily phone surveys that were not sent because of such errors.

### Ethical Considerations

The Multi-Ethnic Study of Atherosclerosis was approved by the University of Washington institutional review board under STUDY00014523, with reliance agreements from participating institutions. All participants provided written informed consent for both the core and ancillary study components. Participants received US $50 compensation for participation in the stress reactivity ancillary study. Data used in this study were deidentified prior to distribution, with HIPAA (Health Insurance Portability and Accountability Act)–defined identifiers removed [20]. Access was limited to approved investigators and governed by MESA data use requirements, including safeguards against participant reidentification, restrictions on disclosure of potentially identifying information, and prohibitions on further data distribution.

### Statistical Analysis

All analyses were descriptive and aimed at evaluating the feasibility of implementing automated EMA phone surveys in a large cohort of older adults. Descriptive statistics were generated to compare the demographics and socioeconomic characteristics of the MESA Exam 7 participants with those of our stress reactivity ancillary study participants. Frequencies and percentages were used to summarize scheduling errors, survey completion rates, and participant-initiated cancellations. All analyses were conducted using SAS (SAS Institute Inc). Data were compiled from REDCap and Smartsheet, and participant-level survey completion logs were used to calculate all outcome measures.

## Results

### Study Enrollment and Data Volume Summary

**Figure 1.**
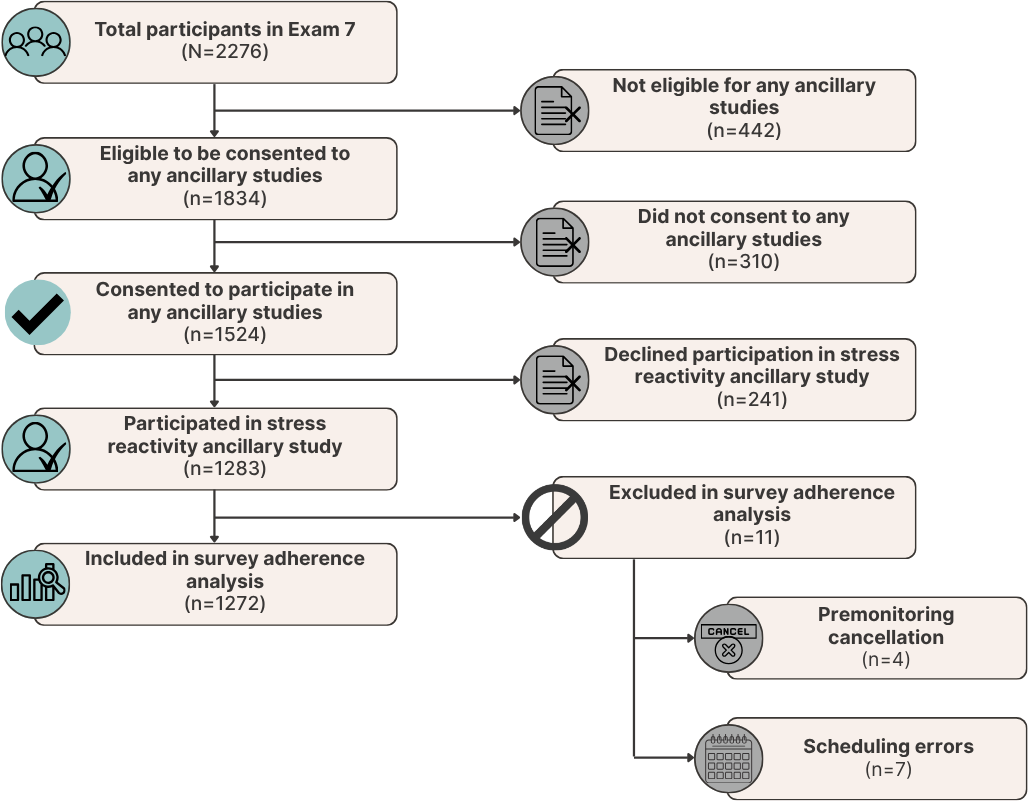
This flowchart illustrates participant recruitment, eligibility, and enrollment from the larger Exam 7 cohort into the stress reactivity ancillary study, and inclusion in the survey adherence analysis.

A total of 1283 participants were enrolled in the MESA Stress Reactivity Ancillary Study from the primary Exam 7 cohort of 2276 individuals. Participant flow from initial Exam 7 completion to final stress reactivity ancillary study enrollment is summarized in Figure 1. While all 1283 participants were included in initial demographic comparisons, the analysis of survey adherence focused on the 1272 individuals who received at least 1 phone survey. A total of 11 participants were excluded from the adherence analysis: 4 individuals who initiated cancellations prior to the monitoring period and 7 individuals who failed to receive any surveys due to administrative scheduling errors. A total of 8835 phone surveys were scheduled across the stress reactivity ancillary study’s enrollment period. This aggregate volume of participants and scheduled surveys serves as the foundation for our evaluation of participant engagement and administrative implementation feasibility.

### Participant Engagement Feasibility: Participant Characteristics and Survey Adherence

A total of 1283 participants were enrolled in the MESA Stress Reactivity Ancillary Study from the larger MESA Exam 7 cohort (N=2276). To evaluate the representativeness of the ancillary study sample, characteristics were compared between enrolled and nonenrolled participants (Table 1). Enrolled participants were significantly younger than those who did not enroll (mean age 75.96, SD 7.07 years vs 79.07, SD 7.75 years; *P*<.001). Significant differences were also observed regarding socioeconomic status. Participants in the ancillary study had higher levels of educational attainment (*P*<.001) and higher annual household incomes (*P*=.01). Specifically, 27.95% (355/1270) of enrolled participants held a graduate degree compared to 24.67% (240/973) of nonenrolled participants, and a higher proportion of the enrolled group reported an income exceeding US $100K (25.06% vs 23.33%). Despite these differences, the enrolled sample remained demographically representative of the larger Exam 7 cohort in terms of gender (*P*=.23) and racial and ethnic distribution (*P*=.88), with similar proportions of African American (338/1283, 26.34%), Chinese (141/1283, 10.99%), Hispanic (282/1283, 21.98%), and non-Hispanic White (522/1283, 40.69%) participants.

**Table 1.** Demographic and socioeconomic characteristics of participants in the MESA^a^ Exam 7 cohort and the subset of participants enrolled in the MESA Stress Reactivity Ancillary Study across the 6 US field sites.

Participant characteristics	MESA Exam 7 participants (N=2276)	MESA Stress Reactivity Ancillary Study participants (enrolled; n=1283)	MESA Stress Reactivity Ancillary Study participants (nonenrolled; n=993)	*P* value^b^
Age (years), mean (SD)	77.3 (7.50)	75.96 (7.07)	79.07 (7.75)	<.001
Sex, n (%)				.23
Female	1249 (54.88)	690 (53.78)	559 (56.29)	
Male	1027 (45.12)	593 (46.22)	434 (43.71)	
Race and ethnicity, n (%)				.88
African American	571 (25.09)	338 (26.34)	233 (23.46)	
Chinese	268 (11.78)	141 (10.99)	127 (12.79)	
Hispanic	516 (22.67)	282 (21.98)	234 (23.56)	
Non-Hispanic White	921 (40.47)	522 (40.69)	399 (40.18)	
Education, n (%)				<.001
High school diploma or less	626 (27.50)	305 (24.02)	321 (32.99)	
Some college, technical, and associate’s degree	589 (25.88)	354 (27.87)	235 (24.15)	
College degree	433 (19.02)	256 (20.16)	177 (18.19)	
Some graduate school or graduate degree	595 (26.14)	355 (27.95)	240 (24.67)	
Income level (US $), n (%)				.01
<$25,000	442 (19.42)	228 (18.67)	214 (23.78)	
$25,000 to <$50,000	535 (23.51)	310 (25.39)	225 (25.00)	
$50,000 to <$100,000	628 (27.59)	377 (30.88)	251 (27.89)	
>$100,000	516 (22.67)	306 (25.06)	210 (23.33)	

a MESA: Multi-Ethnic Study of Atherosclerosis.

b *P* values compare characteristics of the MESA Exam 7 cohort with those enrolled in the stress reactivity ancillary study.

Descriptive analysis of survey completion groups revealed that adherence was largely consistent across demographic and socioeconomic characteristics (Table 2). Age was the only variable significantly associated with survey completion (*P*=.002), with participants in Group 1, the noncompletion group, being significantly older than those in Group 4, the high-adherence group (mean age 79.11 vs 75.74 years). High adherence (5‐7 surveys) remained consistently above 77% across all racial and ethnic subgroups, with the highest rates observed among African American participants (442/520, 85%). However, variation was noted by language. Participants completing surveys in Mandarin (43/56, 76.79%) or Cantonese (22/31, 70.97%) demonstrated lower high-adherence rates than those using English (874/1058, 82.61%) or Spanish (102/126, 80.95%). Completion rates were similar across socioeconomic tiers, with high adherence rates between 78% and 84% for all education and income levels. Noncompletion was uncommon across all subgroups, generally ranging between 4% and 10%.

**Table 2.** Distribution of daily phone survey completion groups by demographic and socioeconomic characteristics among participants in the MESA^a^ Stress Reactivity Ancillary Study.

		Number of phone surveys completed			
Participant characteristics	Overall^b^ (N=1272)	Group 1^c^: 0 surveys (n=66)	Group 2^c^: 1‐2 surveys (n=50)	Group 3^c^: 3‐4 surveys (n=115)	Group 4^c^: 5‐7 surveys (n=1041)
Age (years)^d^, mean (SD)	75.92 (7.05)	79.11 (7.92) ^d^	76.42 (7.09)	75.51 (7.58)	75.74 (6.89)
Sex, n (%)					
Female	684 (53.73)	33 (4.82)	28 (4.09)	56 (8.19)	567 (82.89)
Male	588 (46.23)	33 (5.61)	22 (3.74)	59 (10.03)	474 (80.61)
Race and ethnicity, n (%)					
African American	520 (40.85)	22 (4.23)	20 (3.85)	36 (6.92)	442 (85.00)
Chinese	135 (10.60)	10 (7.41)	6 (4.44)	15 (11.11)	104 (77.04)
Hispanic	337 (26.47)	17 (5.04)	13 (3.86)	35 (10.39)	272 (80.71)
Non-Hispanic White	280 (22.01)	17 (6.07)	11 (3.93)	29 (10.36)	223 (79.64)
Language of call, n (%)					
English	1058 (83.18)	50 (4.73)	39 (3.69)	95 (8.98)	874 (82.61)
Spanish	126 (9.91)	9 (7.14)	7 (5.56)	8 (6.35)	102 (80.95)
Chinese (Mandarin)	56 (4.40)	4 (7.14)	2 (3.57)	7 (12.50)	43 (76.79)
Chinese (Cantonese)	31 (2.44)	3 (9.68)	2 (6.45)	4 (12.90)	22 (70.97)
Education, n (%)					
High school diploma or less	300 (23.81)	13 (4.33)	8 (2.67)	27 (9.00)	252 (84.00)
Some college, technical, and associate’s degree	351 (27.88)	20 (5.70)	18 (5.13)	38 (10.83)	275 (78.35)
College degree	255 (20.24)	15 (5.88)	8 (3.14)	22 (8.63)	210 (82.35)
Some graduate school or graduate degree	353 (28.02)	18 (5.10)	15 (4.25)	28 (7.93)	292 (82.72)
Income level (US $), n (%)					
<$25,000	224 (18.48)	15 (6.70)	7 (3.13)	18 (8.04)	184 (82.14)
$25,000 to <$50,000	308 (25.41)	13 (4.22)	13 (4.22)	33 (10.71)	249 (80.84)
$50,000 to <$100,000	374 (30.88)	18 (4.81)	13 (3.48)	33 (8.82)	310 (82.89)
>$100,000	305 (25.17)	15 (4.92)	13 (4.26)	24 (7.87)	253 (82.95)

a MESA: Multi-Ethnic Study of Atherosclerosis.

b Values are presented as n (%). Percentages in the Overall column are column percentages.

c Values are presented as n (%). Percentages in Groups 1‐4 are row percentages.

d Significant at the .05 level.

Beyond demographic and socioeconomic trends, we also assessed the practical experience of survey completion. Among the 1272 participants who received at least 1 phone survey during the 7-day monitoring period, the median survey completion time was 3 (IQR 2‐4) minutes, which was consistent with the anticipated duration. However, we evaluated extended durations as a proxy for participant burden and tolerability. While the phone surveys were designed to be less than 5 minutes, 2371 of the total 8835 (26.84%) surveys lasted 5 minutes or longer. This was a common experience among the participants in the survey adherence analysis, with 73.19% (931/1272) of participants completing at least 1 survey that met this threshold. For these phone surveys, the mean completion time was approximately 7 (SD 5.03) minutes. Despite the increased time commitment, these participants demonstrated high adherence, completing a median of 6 (IQR 3-7) surveys.

More extreme durations were rare but followed a distinct demographic pattern. Surveys lasting 10 minutes or more accounted for 2.60% (230/8835) of total phone surveys and occurred among 15.25% (194/1272) of participants. Phone surveys exceeding 20 minutes were exceptionally infrequent, representing only 0.62% (53/8835) of all surveys. We observed that participants who required more time to complete the phone surveys tended to be older than the average participant, with the mean age increasing incrementally alongside survey duration from 76.09 (SD 7.01) years in the 5-minute or longer category to 77.73 (SD 7.31) years in the 20-minute or longer category. Notably, while participants experiencing these longer durations were more likely to miss at least 1 individual phone survey, they consistently completed most of their scheduled phone surveys across the 7-day monitoring period, maintaining a median of 6 completed surveys across both duration categories (IQR for over 10 minutes: 5-7; IQR for over 20 minutes: 4-7).

### Participant Engagement Feasibility: Cancellations

Participant-initiated cancellations occurred both before and during the monitoring period. Of the 1283 participants enrolled in the stress reactivity ancillary study, premonitoring cancellations only accounted for 0.31% (4/1283) of participants. These early withdrawals were primarily associated with an inability to comply with other broader aspects of the ancillary study rather than issues specific to the automated phone system. During monitoring, cancellations accounted for 1.65% (21/1272) of participants who received at least 1 phone survey. Of these individuals, 23.81% (5/21) had completed at least 1 survey before deciding to discontinue.

### Administrative Implementation Feasibility

To evaluate administrative implementation feasibility, we assessed operational accuracy by identifying scheduling errors that disrupted the intended 7-day survey sequence. Among the 1283 participants enrolled in the stress reactivity ancillary study, only 7 (0.55%) individuals were affected by scheduling errors that prevented their phone surveys from being initiated. As a result, these participants did not receive any of the daily phone surveys during the 7-day monitoring period. An additional 17 (1.33%) participants received fewer than the 7 intended phone surveys because of scheduling errors during the initial scheduling process at the clinic visit. Additionally, only 0.42% (37/8835) were not delivered because of scheduling errors. These findings indicate that scheduling errors affected only a small proportion of participants and expected surveys, supporting the administrative implementation feasibility of the automated phone-based daily surveys across a large, multisite research infrastructure.

## Discussion

### Principal Findings

By evaluating both participant engagement and administrative implementation, this study supports the feasibility of automated daily phone surveys for collecting real-time data in a large, geographically diverse cohort of older adults. Comparing the ancillary study sample with the broader MESA Exam 7 cohort provided important context for interpreting the generalizability of these feasibility findings. The lack of significant differences in gender distribution (*P*=.23) and racial and ethnic composition (*P*=.88) suggests that the ancillary study sample was broadly similar to the Exam 7 cohort across these demographic characteristics. However, participants enrolled in the ancillary study were younger (*P*<.001) and had higher levels of education (*P*<.001) and household income (*P*=.01) than those who were not enrolled. Because age was the only participant characteristic significantly associated with survey completion (*P*=.002), the high adherence rate of 81.84% observed in this study may be higher than what would be expected in older cohorts or in populations with fewer socioeconomic resources. Therefore, while our findings support the feasibility of automated daily phone surveys, additional tailoring may be needed to support consistent engagement among older adults who may face greater barriers to participation.

Participant engagement remained high, with 81.84% (1041/1272) of the participants who received at least 1 survey demonstrating high adherence by completing 5‐7 surveys. Statistical analysis revealed that age was the only demographic variable significantly associated with survey completion (*P*=.002). Although adherence was consistently high across racial and ethnic groups, descriptive variation was observed by survey language, as Mandarin- and Cantonese-speaking participants showed slightly lower rates of high adherence compared to English- and Spanish-speaking participants. The study used translated scripts but did not implement additional system-level modifications specific to tonal languages. Because formal feedback was not systematically collected from Mandarin- and Cantonese-speaking participants, we cannot determine whether language delivery, automated voice quality, cultural adaptation, or other factors contributed to these differences. These findings suggest that future automated daily phone surveys may benefit from additional usability testing and language-specific tailoring for individuals who speak other languages. At the same time, offering the phone surveys in English, Spanish, Mandarin, and Cantonese according to participant preference may have contributed to the enhanced engagement across this cohort.

This feasibility study also offers insights regarding participant burden and tolerability. While surveys were designed to take no more than 5 minutes, 26.84% (2371/8835) of all phone surveys exceeded this duration. Despite these extended survey durations, adherence remained high: participants who experienced at least 1 survey lasting longer than 5 minutes still completed a median of 6 phone (IQR 3-7) surveys. These findings suggest that the automated phone survey protocol was generally tolerable for older adults, even when individual surveys exceeded the intended duration. The simplified response format, consisting primarily of Yes or No keypad entries and brief numeric scales, may have contributed to the high adherence observed.

A small subset of participants in Group 1 provided feedback on why they did not respond to any or missed the phone surveys: 8 participants reported never receiving the phone surveys, 5 cited hearing difficulties, and 4 mentioned phone-related issues such as a disconnected number or accidentally blocking the study line. These observations suggest that noncompletion among this subgroup should be explored further to uncover specific barriers to participation and to determine whether additional tailoring in the automated phone-based survey approach could enhance engagement and accessibility.

The use of premonitoring and during monitoring cancellation metrics provided a nuanced view of study attrition. Both forms of participant-initiated cancellation were rare, with 4 premonitoring cancellations and 21 during monitoring cancellations, totaling 25 participants, or 1.95% (25/1283) of the 1283 ancillary study participants. A small subset of participants provided reasons for canceling the phone surveys. Most premonitoring withdrawals were associated with an inability to comply with broader study components rather than the phone surveys themselves, even though participants were encouraged to continue the daily phone surveys regardless of their participation in other study components. Other individuals reported finding the automated calls a nuisance, preferring live interviewer calls for privacy, or feeling stressed while waiting for the scheduled automated phone calls. Despite these instances, the low overall rate of attrition supports the high acceptability of the automated daily phone surveys within an older adult population.

Beyond participant-facing features, the protocol optimized administrative implementation feasibility and staff sustainability by automating the initial scheduling process, which significantly reduced the daily administrative burden on field center staff and provided a notable advantage for large-scale studies where coordination across multiple sites is complex. This reduction in daily tasks allowed staff to prioritize proactive participant follow-up, troubleshoot technical issues, and support overall protocol adherence. Together, these findings help fill an important gap in the EMA literature and support the feasibility of using automated phone surveys in a large, multisite population of older adults.

### Limitations

Despite the promising results, several limitations should be considered. First, although the majority of participants completed most or all of the surveys, a small subset either completed none or requested early cancellation. These individuals may have experienced barriers such as limited comfort with phone technology, hearing impairments, or other unmeasured factors. However, because our protocol did not include a formal process for collecting feedback from participants who were disengaged, we were unable to determine the reasons for noncompletion. Incorporating brief follow-up interviews or satisfaction surveys could provide valuable insights for future research. Additionally, we did not assess participants’ perceptions of the survey experience, such as satisfaction or perceived burden. These perspectives are essential for interpreting adherence and for refining EMA protocols to maximize feasibility and acceptability among older adults. Finally, although the delivery of phone surveys was fully automated, the initial scheduling process relied on manual entry by field staff. While errors were rare, this step remains a potential source to further streamline workflows. Future efforts might explore opportunities to automate or standardize scheduling processes, informed by field staff feedback, to further minimize the risk of human error, especially in multisite studies where coordination is complex.

### Conclusion

Our findings suggest that daily EMA surveys by phone can be both practical and scalable for use in large, multisite studies involving older adults. High adherence rates, as well as low cancellation and operational error, suggest this approach is not only feasible but well-tolerated in a real-world research setting. These results provide a promising model for incorporating EMA into aging research. Future studies should explore strategies to improve accessibility among subgroups with lower adherence (eg, due to hearing problems) and evaluate the validity and predictive value of phone-based EMA data. As EMA continues to evolve as a tool for real-time data capture, our findings offer actionable insights for designing and deploying EMA in older adult populations, with implications for broader adoption in multisite health research.

## Abbreviations

EMA: ecological momentary assessment
HIPAA: Health Insurance Portability and Accountability Act
MESA: Multi-Ethnic Study of Atherosclerosis

## References

[1] Shiffman S, Stone AA, Hufford MR (2008). Ecological momentary assessment. Annu Rev Clin Psychol.

[2] Burke LE, Shiffman S, Music E (2017). Ecological momentary assessment in behavioral research: addressing technological and human participant challenges. J Med Internet Res.

[3] Wrzus C, Neubauer AB (2023). Ecological momentary assessment: a meta-analysis on designs, samples, and compliance across research fields. Assessment.

[4] Businelle MS, Hébert ET, Shi D (2024). Investigating best practices for ecological momentary assessment: nationwide factorial experiment. J Med Internet Res.

[5] Smyth JM, Jones DR, Wen CKF, Materia FT, Schneider S, Stone A (2021). Influence of ecological momentary assessment study design features on reported willingness to participate and perceptions of potential research studies: an experimental study. BMJ Open.

[6] Compernolle S, Vetrovsky T, Maes I (2024). Older adults’ compliance with mobile ecological momentary assessments in behavioral nutrition and physical activity research: pooled results of four intensive longitudinal studies and recommendations for future research. Int J Behav Nutr Phys Act.

[7] Ramsey AT, Wetherell JL, Depp C, Dixon D, Lenze E (2016). Feasibility and acceptability of smartphone assessment in older adults with cognitive and emotional difficulties. J Technol Hum Serv.

[8] Nam S, Dunton GF, Ordway MR (2020). Feasibility and acceptability of intensive, real-time biobehavioral data collection using ecological momentary assessment, salivary biomarkers, and accelerometers among middle-aged African Americans. Res Nurs Health.

[9] Crawford JL, English T, Braver TS (2022). Incorporating ecological momentary assessment into multimethod investigations of cognitive aging: promise and practical considerations. Psychol Aging.

[10] Cain AE, Depp CA, Jeste DV (2009). Ecological momentary assessment in aging research: a critical review. J Psychiatr Res.

[11] Maher JP, Rebar AL, Dunton GF (2018). Ecological momentary assessment is a feasible and valid methodological tool to measure older adults’ physical activity and sedentary behavior. Front Psychol.

[12] Moore RC, Kaufmann CN, Rooney AS (2017). Feasibility and acceptability of ecological momentary assessment of daily functioning among older adults with HIV. Am J Geriatr Psychiatry.

[13] Rullier L, Atzeni T, Husky M (2014). Daily life functioning of community-dwelling elderly couples: an investigation of the feasibility and validity of Ecological Momentary Assessment. Int J Methods Psychiatr Res.

[14] Stone AA, Schneider S, Smyth JM (2023). Evaluation of pressing issues in Ecological Momentary Assessment. Annu Rev Clin Psychol.

[15] Charness N, Boot WR (2009). Aging and information technology use: potential and barriers. Curr Dir Psychol Sci.

[16] Smith A (2014). Older adults and technology use: usage and adoption. http://www.pewinternet.org/2014/04/03/usage-and-adoption/.

[17] Yao L, Yang Y, Wang Z, Pan X, Xu L (2023). Compliance with ecological momentary assessment programmes in the elderly: a systematic review and meta-analysis. BMJ Open.

[18] Bild DE, Bluemke DA, Burke GL (2002). Multi-Ethnic Study of Atherosclerosis: objectives and design. Am J Epidemiol.

[19] (2025). The Multi-Ethnic Study of Atherosclerosis. MESA.

[20] (2026). Overview and protocol. MESA.

[21] Almeida DM, Wethington E, Kessler RC (2002). The daily inventory of stressful events. Assessment.

[22] Gupta G (2023). Improve participant engagement in redcap healthcare research with Twilio SMS & voice. Twilio.

[23] (2019). BioLINCC - biologic specimen and data repository information coordinating center. National Institutes of Health.

[24] (2026). Database of genotypes and phenotypes. National Institutes of Health.

